# X-linked hyper IgM syndrome with severe eosinophilia: a case report and review of the literature

**DOI:** 10.1186/s12887-022-03251-z

**Published:** 2022-04-04

**Authors:** He Li, Yang Cao, Jijun Ma, Chongwei Li

**Affiliations:** Department of Rheumatology & Immunology,Tianjin Children’s Hospital, No. 238 Longyan Road, Beichen District, Tianjin, China

**Keywords:** X-linked hyper IgM syndrome, Severe eosinophilia, Interstitial lung disease, Pneumocystis jirovecii pneumonia

## Abstract

**Background:**

Hyper IgM syndromes (HIGMS) are a group of rare primary immunodeficiency disorders. There are limited reports about HIGMS combined with severe eosinophilia.

**Case presentation:**

In this report, we described a 2-year-old boy with chronic cough and symptoms of hypoxia. Lung computed tomography (CT) scan showed that diffuse ground-glass changes and eosinophils in peripheral blood increased significantly. Subsequent tests revealed a notable decrease in serum IgG and IgA. The lymphocyte subgroup classification was basically normal. Pneumocystis jirovecii were detected from the bronchoalveolar lavage fluid (BALF) of the patient by metagenomic next-generation sequencing (mNGS). After treatments of caspofungin combined with sulfamethoxazole, intravenous immunoglobulin (IVIG) replacement and anti-inflammatory steroid, the clinical symptoms and pulmonary imaging noticeably improved. The absolute eosinophil count (AEC) also returned to normal range. X-linked hyper IgM syndrome was confirmed by gene test. Two months after the diagnosis, the patient underwent allogeneic stem cell transplantation (HSCT) and has recovered well.

**Conclusions:**

Children with HIGMS are prone to opportunistic infections such as Pneumocystis jirovecii pneumonia (PJP). Diffuse interstitial lung disease and hypoglobulinemia in a young child predict the diagnosis of a primary immunodeficiency (PID). mNGS has obvious advantages for obtaining etiological diagnosis of children with PIDs. Severe eosinophilia is rarely reported in this kind of PIDs. Considering literature review and the corresponding reaction to steroid, we proposed that eosinophilia in HIGMS might be related to infections. Steroid therapy can quickly relieve eosinophilia but is easy to rebound if the reduction is too fast. Once the diagnosis of HIGMS is confirmed, the earlier the HSCT, the better the prognosis.

## Background

Hyper IgM syndromes (HIGMS) are a group of rare primary immune deficiency diseases first reported by Rosen et al. in 1961. Six main subtypes of HIGMS have be characterized according to different genetic defects [1]. The X-linked hyper IgM syndrome (XHIGM, HIGM1) is the most common type and is caused by mutations in the CD40 ligand gene. The clinical feature is recurrent infections, particularly the high incidence of opportunistic pathogens. Patients with HIGMS have decreased concentrations of serum IgG, IgA and IgE, normal or elevated IgM and normal peripheral blood B-lymphocyte. XHIGM is often accompanied with neutropenia. Pneumocytis jirovecii(PJ) is the most common pathogen causing pneumonia in XHIGM patients [2, 3]. Here, we present a 2-year-old XHIGM patient with severe eosinophilia, which is rarely reported. In addition, we reviewed published reports to discuss the main clinical findings, the diagnostic and therapeutic approach for this case.

## Case presentation

**Fig. 1:**
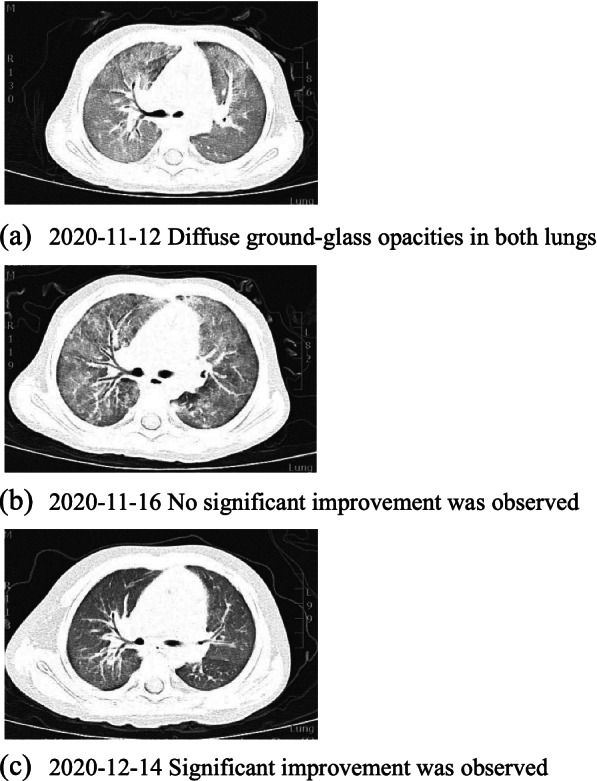
Comparison of chest computed tomography images during hospitalization. **a **2020-11-12 Diffuse ground-glass opacities in both lungs. **b** 2020-11-16 No significant improvement was observed. **c** 2020-12-14 Significant improvement was observed

**Fig. 2:**
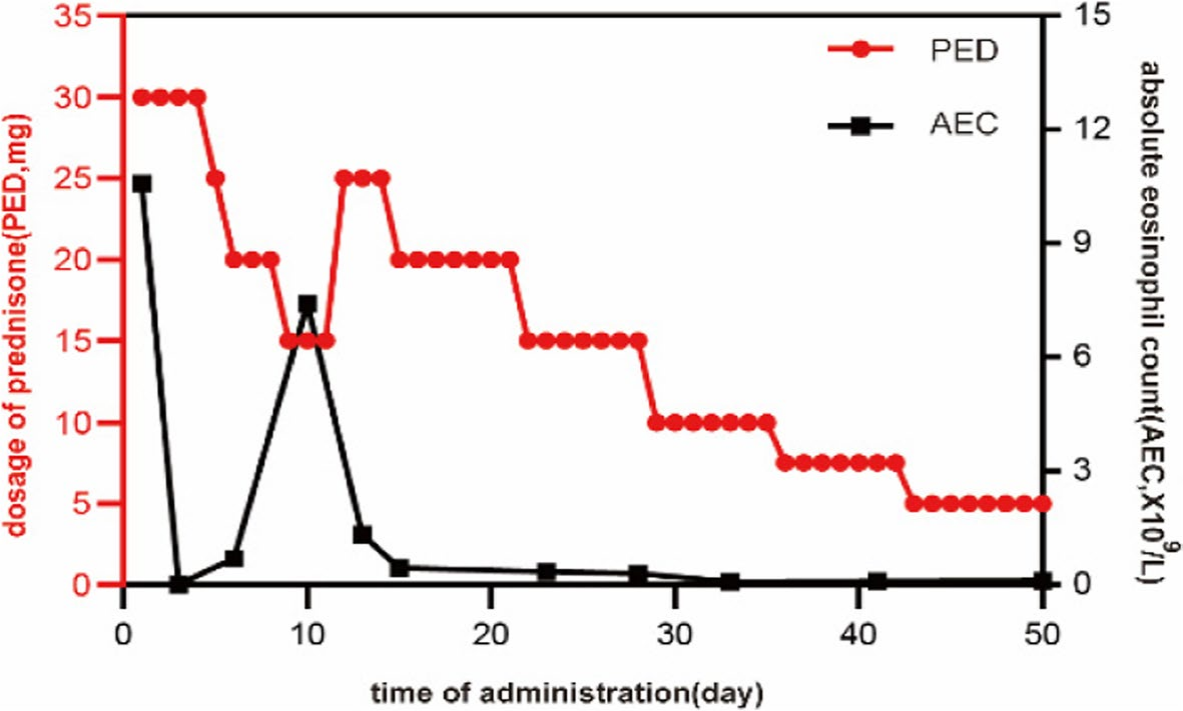
Relationship between steroid and AEC

**Fig. 3:**
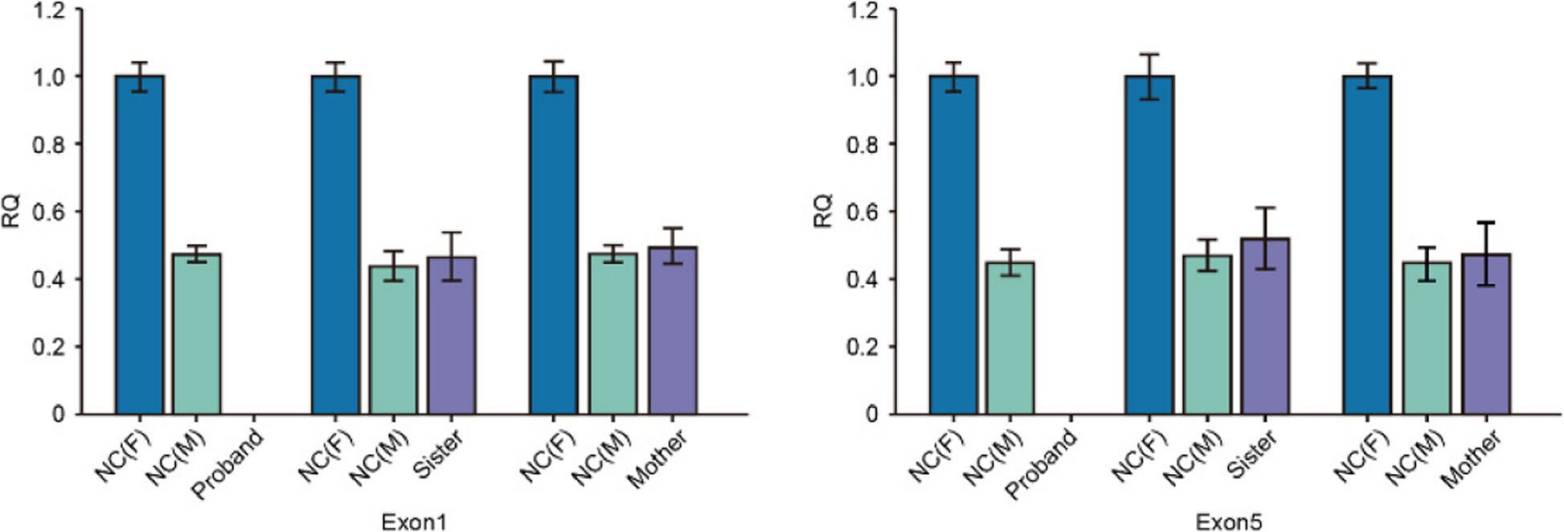
Expression of exon1 and exon 5 of CD40L gene in proband, his sister and mother

On November 12th, 2020, a 2-year-old boy with one-month chronic cough was admitted to Tianjin Children’s Hospital. His cough was mild with less sputum. No obvious wheezing, dyspnea or fever was observed. Oral antitussive medicine was taken at home but did not work. Afterwards, the patient was sent to local hospital to take a two-day intravenous administration of cefotaxime sodium. Peripheral blood test revealed a clear increase of white blood cell count (WBC23.9 × 10^9^/L) with eosinophilia (58.91%, AEC14.1 × 10^9^/L) and neutropenia (3.32%, absolute neutrophil count 0.8 × 10^9^/L). The patient was admitted for further diagnosis and treatment by Department of Immunology. The parents claimed that the boy had BCG lymphadenitis (self-healing) previously and has been suffering from upper respiratory infections four to five times a year when peripheral blood tests indicated a decrease in granulocytes. His family history included a deceased elder brother who had BCG lymphadenitis (incision and drainage) and died of cyanosis at the age of 11 months. His test results revealed that C-reactive protein (CRP), hemoglobin and platelet approached the normal limits, while neutrophil (7%, normal range 45–77%, ANC 1.27 × 10^9^/L) decreased and eosinophil (46%, normal range 0.5-5%, AEC 10.58 × 10^9^/L) elevated notably. Biochemical indicators were basically normal with a slight increase in lactate dehydrogenase (LDH) 490U/L (normal range 120–300). IgA (0.05 g/L, normal range 0.2-1.0 g/L), IgG (0.07 g/L, normal range 4.53–9.16 g/L) and IgE (< 0.1 IU/ml, normal range 0-100 IU/ml) all decreased whereas IgM was in normal range (0.7 g/L, normal range 0.19–1.46 g/L). Lung computed tomography (CT) scan showed multiple diffuse ground-glass opacities and patchy consolidation in both lungs (Fig. 1a). The admission diagnosis was severe pneumonia with neutropenia and eosinophilia, and PID was suspected. The initial anti-infective therapy included cefoperazone-sulbactam, trimethoprim-sulfamethoxazole (SMZCo) and voriconazole. The patient also received a 2 g/Kg intravenous immunoglobulin (IVIG) replacement, an anti-inflammatory of 2 mg/kg/d prednisone and oxygen inhalation with respiratory management. Lymphocyte subsets showed CD19^+^ 44.1% (normal range 17–41%), CD3^+^ 52.5% (normal range 39–73%), CD3^+^CD8^+^ 21.28% (normal range 11–32%), CD3^+^CD4^+^ 30.3% (normal range 25–50%) and CD16^+^CD56^+^ 2.03% (normal range 3–16%). Blood pathogen tests on EBV-DNA, CMVPP65, (1,3)-β-d-glucan test, glactomannan (GM), tuberculosis, cryptococcus capsular antigen, antibody test for parasites including schistosoma, paragonimus, cysticercus, trichinella spiralis, liver fluke, sparganum and hydatid were all negative. Peripheral blood was tested again after three days, showing a normal level of WBC (5.78 × 10^9^/L) and eosinophils (0.2%, AEC 0.01 × 10^9^/L). Cefoperazone sulbactam was discontinued and the dosage of prednisone was reduced quickly. No significant improvement was observed on lung CT after five days in hospital (Fig. 1b). The fiberoptic bronchoscopy was performed. Cytology results of the BALF were neutrophil 8%, eosinophil 5%, lymphocyte 6%, macrophage 78% and epithelium 3%. mNGS of the BALF showed Pneumocystis jirovecii (PJ) infection. Voriconazole was discontinued, caspofungin was used together with SMZCo to treat PJP. When prednisone was reduced to 15 mg/d, the count of WBC (17.76 × 10^9^/L) and eosinophils (36%, AEC 6.39 × 10^9^/L) rebounded. After we adjusted the dosage of prednisone and reduced it gradually, AEC maintained in a normal range (Fig. 2). The child was discharged 15 days after the initial treatment and continued to take SMZCo and prednisone. One month after discharge, he was hospitalized again for IVIG replacement therapy. The lung CT scan was notably improved (Fig. 1c). With the informed consent from the patient’s family, venous blood was collected from four members of the family and sent to Beijing Genomics Institute (BGI) for whole exome sequencing (WES) test. The results revealed a large deletion in the 1st to 5th exon of CD40L gene in the patient, and thus XHIGM was confirmed. Sanger sequencing was used to verify the CD40L gene fragment in peripheral blood DNA of the other family members, which indicated a heterozygous mutation for his mother and elder sister (Fig. 3). His father’s genotype is normal. The patient underwent a successful HSCT two months after diagnosis in another hospital. The donor was the sibling sister, and HLA matching was 9/10. After HSCT, the peripheral blood test showed WBC5.13 × 10^9^/L, ANC2.81 × 10^9^/L, AEC0.1 × 10^9^/L, which were all within normal range. In addition to the normal IgE (5 IU/mL, normal range 0-165IU/mL), IgG was pronouncedly elevated to a normal level (1020 mg/dl, normal range 500-1300 mg/dl) with slight decreases of IgA (6.67 mg/dl, normal range 22-220 mg/dl) and IgM (10.4 mg/dl, normal range 43-163 mg/dl). The patient was in a good condition after three months follow-up with 99.93% donor chimerism. Mycophenolate mofetil and cyclosporine were used for graft-versus-host disease (GvHD) prophylaxis.

## Discussion and conclusion

The X-linked form of HIGMS (XHIGM) is most common and accounts for about 65–70% of all cases with the HIGM phenotypes. The gene connected with XHIGM was mapped to the long arm of the X-chromosome (Xq26.3 − 27.1) including five exons. The point mutations are the most common source of genetic variation, and missense mutations are the most frequent. CD40L gene mutation was confirmed by WES in this case, which showed a large deletion about 59 kb from 135730409 to 135789189, which encompasses exon1 to exon5. To our knowledge, only three cases of the same gene defect have been reported in the world to date. There was no significant difference in clinical phenotype between large fragment deletions and point mutations [4].

CD40L-CD40 interaction is the first step in B cell stimulation for class switch recombination (CSR) and somatic hyper mutation (SHM). It influences the interactions between CD4 + T cells and antigen presenting cells (APCs). CD40L gene mutation plays an important role in the pathogenesis of XHIGM, which in known as a defective geometrical folding of CD40L that prevents the CD40L/CD40 binding [5]. Therefore, XHIGM is a combined immunodeficiency disease. The main clinical manifestations include recurrent infections (lung and gastrointestinal tract) and high incidence of opportunistic infections (Pneumocystis jirovecii, Histoplasma capsulatum and Cryptosporidium), particularly within two years of birth [6]. The clinical features in this case were dry cough and shortness of breath. There was no obvious rale in the lung auscultation, but the lung imaging changes were severe, suggesting diffuse interstitial lung disease (ILD). The American Thoracic Society guidelines for classification, evaluation and management of childhood ILD pointed out that infections can be the cause of ILD and all children younger than two years old should be screened for immunodeficiency [7]. Common pathogens causing ILD include virus, mycoplasma, Legionella and PJ. Considering the background of immune deficiency and imaging findings, it was highly suspected that the patient had PJP. However, no positive result was obtained using Gomori methenamine silver (GMS) staining in the BALF. PJ infection was further confirmed by mNGS. It is difficult to detect PJ in early stage. GMS in sputum or BALF is generally accepted as the staining method for the diagnosis of PJP. However, the detection rate is very low. In comparison, mNGS, with its high sensitivity, accuracy and simplicity, is of great value for identifying the etiology in children with immunodeficiency [8].

Eosinophils, originating from hematopoietic stem cells, are a type of bone marrow cell in peripheral blood. Its development, migration and function are closely regulated by networks of transcription factors, growth factors, cytokines and chemokines. Normally, the absolute count of eosinophils in peripheral blood is less than 0.5 × 10^9^/L. When the level increases markedly to more than 5 × 10^9^/L, it is defined as severe eosinophilia, and the probability of tissue damage increases pronouncedly [9]. The patient in this case showed abnormal increase of eosinophil ratio in his peripheral blood and BALF. Peripheral eosinophilia can be divided into categories of primary, secondary and idiopathic eosinophilia. Allergy, parasite infection, drug reaction, autoimmune or inflammatory disease and tumor are all causes of peripheral eosinophilia [10]. Immune deficiency should be considered particularly in children [11]. The common PIDs causing eosinophilia include hyper IgE syndrome (HIES), dedicator of cytokinesis 8 (DOCK8) deficiency, phosphoglucomutase3 (PGM3) deficiency, adenosine deaminase-severe combined immunodeficiency (ADA-SCID), Omenn syndrome, Wiskott-Aldrich syndrome (WAS) and so on. The mechanism driving the eosinophilia is unclear. These PIDs are characterized by increased production of cytokines secreted by Th2 cell, such as interleukin 5 (IL-5), which plays an essential role in proliferation, differentiation, maturation and release of eosinophils [12]. Meanwhile, these types of PIDs are often associated with clinical manifestations of atopy and increased IgE levels, which was not found in HIGMS. Throughout the literature review, we found only seven case reports about HIGMS with eosinophilia [13–19]. In the reports, most of the patients were infected with parasites (Cryptosporidium) or fungi (Pneumocystis jirovecii and Penicillium marneffei). We speculate that eosinophilia in HIGMS may be related to the infections. Microbial antigens can stimulate TH2 cells and induces cytokines such as IL-5, resulting in highly increased numbers of eosinophils. An Italian girl in the literature had eosinophilia when suffering from a cryptosporidium infection at 7-year-old, however, eosinophilia did not occur during her PJ infections at 4-month-old and 2-year-old [15]. Therefore, further study on the mechanism of eosinophilia is necessary. In this case, our experience shows that the reduction of steroid should not be too quick, a two or three-month duration of administration is recommended.

For the treatment of XHIGM, although IVIG replacement, application of granulocyte colony-stimulating factor (G-CSF) and preventive antibiotic therapy are effective, life-threatening infection can still lead to high mortality and the incidence of autoimmune diseases and malignant tumors is still high. Therefore, bone marrow transplantation or HSCT should be carried out as soon as possible [20].

## Data Availability

Not applicable.

## Abbreviations

HIGMS: Hyper IgM syndromes
mNGS: metagenomic next-generation sequencing
PJP: Pneumocystis jirovecii pneumonia
ILD: interstitial lung disease
XHIGM: X-linked hyper IgM syndrome
SMZCo: trimethoprim-sulfamethoxazole

## References

[1] Griffin DD, Dolen WK. B cell disorders in children: Part II. Curr Allergy Asthma Rep. 2020;20(11):64. 10.1007/s11882-020-00963-z 32821980

[2] Fuleihan R, Ramesh N, Loh R, Jabara H, Rosen FS, Chatila T (1993). Defective expression of the CD40 ligand in x-chromosome-linked immunoglobulin deficiency with normal or elevated IgM. Proc Natl Acad Sci U S A.

[3] Etzioni A, Ochs HD (2004). The hyper IgM syndrome - An evolving story. Pediatr Res.

[4] Zhu X, Xia Y, Yang J (2019). Hyper-IgM syndrome caused by copy number variation:One case report and literature review. Chin J Evid Based Pediatr.

[5] Yazdani R, Fekrvand S, Shahkarami S, Azizi G, Moazzami B, Abolhassani H (2019). The hyper IgM syndromes: Epidemiology, pathogenesis, clinical manifestations, diagnosis and management. Clin Immunol.

[6] Picard C, Gaspar H, Al-Herz W, Bousfiha A, Casanova JL, Chatila T (2018). International Union of Immunological Societies: 2017 Primary Immunodeficiency Diseases Committee Report on Inborn Errors of Immunity. J Clin Immunol.

[7] Kurland G, Deterding RR, Hagood JS, Young LR, Brody AS, Castile RG (2013). An Official American Thoracic Society Clinical Practice Guideline: Classification, Evaluation, and Management of Childhood Interstitial Lung Disease in Infancy. Am J Respir Crit Care Med.

[8] Gu P, Xu S, Jiang X, Zhou Y, Zhou Y, Li Z (2020). Diagnosis of pneumocystis pneumonia by metagenomic next-generation sequencing in patients with kidney disease. Chin J Nephrol Dialysis Transplantation.

[9] Shomali W, Gotlib J (2019). World Health Organization-defined eosinophilic disorders: 2019 update on diagnosis, risk stratification, and management. Am J Hematol.

[10] Valent P, Klion AD, Horny HP, Roufosse F, Gotlib J, Weller PF (2012). Contemporary consensus proposal on criteria and classification of eosinophilic disorders and related syndromes. J Allergy Clin Immunol.

[11] Kelli W, Williams, Joshua D, Milner, Alexandra F, Freeman. Eosinophilia Associated with Disorders of Immune Deficiency or Immune Dysregulation. Immunol Allergy Clin North Am. 2015;35(3):523–44. 10.1016/j.iac.2015.05.004 PMC4688016 26209898

[12] Fulkerson PC, Schollaert KL, Bouffi C, Rothenberg ME (2014). IL-5 triggers acooperative cytokine network that promotes eosinophil precursor maturation. J Immunol.

[13] Jo EK, Kim HS, Lee MY, Iseki M, Lee JH, Song CH (2002). X-linked hyper-IgM syndrome associated with Cryptosporidium parvum and Cryptococcus neoformans infections: The first case with molecular diagnosis in Korea. J Korean Med Sci.

[14] Kutukculer N, Moratto D, Aydinok Y, Lougaris V, Aksoylar S, Plebani A (2003). Disseminated Cryptosporidium infection in an infant with hyper-IgM syndrome caused by CD40 deficiency. J Pediatr.

[15] Lougaris V, Badolato R, Ferrari S, Plebani A (2005). Hyper immunoglobulin M syndrome due to CD40 deficiency: clinical, molecular, and immunological features. Immunol Rev.

[16] Joseph L, Rudensky B, Cohen S, Goldberg S, Schlesinger Y, Picard E (2005). Eosinophilia, pneumonia and hypogammaglobulinemia. Pediatr Infect Disease J.

[17] Merchant RH, Ahmed J, Ahmed N, Picard C (2014). Type I Hyper IgM Syndrome with Novel Mutation from India. Indian J Pediatr.

[18] Guo L, Chen B, Xu B, Lu MP, Ning BT, Chen ZJ (2015). X-linked hyper-IgM syndrome with eosinophilia in a male child: A case report. Experimental and Therapeutic Medicine.

[19] Liu D, Zhong L, Li Y, Chen M (2016). Recurrent fever,hepatosplenomegaly and eosinophilia in a boy. Chin J Contemp Pediatr.

[20] Heimall J, Puck J, Buckley R, Fleisher TA, Gennery AR, Neven B, et al. Current Knowledge and Priorities for Future Research in Late Effects after Hematopoietic Stem Cell Transplantation (HCT) for Severe Combined Immunodeficiency Patients: A Consensus Statement from the Second Pediatric Blood and Marrow Transplant Consortium International Conference on Late Effects after Pediatric HCT. Biol Blood Marrow Transpl. 2017;23:379 – 87. 10.1016/j.bbmt.2016.12.619 PMC5659271 28068510

